# EDR Peptide: Possible Mechanism of Gene Expression and Protein Synthesis Regulation Involved in the Pathogenesis of Alzheimer’s Disease

**DOI:** 10.3390/molecules26010159

**Published:** 2020-12-31

**Authors:** Vladimir Khavinson, Natalia Linkova, Ekaterina Kozhevnikova, Svetlana Trofimova

**Affiliations:** 1 Department of Biogerontology, Saint Petersburg Institute of Bioregulation and Gerontology, 197110 Saint Petersburg, Russia; khavinson@gerontology.ru (V.K.); katena_94@list.ru (E.K.); dr.s.trofimova@gmail.com (S.T.); 2 Group of Peptide Regulation of Aging, Pavlov Institute of Physiology of the Russian Academy of Sciences, 199004 Saint Petersburg, Russia

**Keywords:** tripeptide, neuroprotection, MAPK, apoptosis, SOD2, GPX1, PPARA, PPARG, serotonin, calmodulin

## Abstract

The EDR peptide (Glu-Asp-Arg) has been previously established to possess neuroprotective properties. It activates gene expression and synthesis of proteins, involved in maintaining the neuronal functional activity, and reduces the intensity of their apoptosis in in vitro and in vivo studies. The EDR peptide interferes with the elimination of dendritic spines in neuronal cultures obtained from mice with Alzheimer’s (AD) and Huntington’s diseases. The tripeptide promotes the activation of the antioxidant enzyme synthesis in the culture of cerebellum neurons in rats. The EDR peptide normalizes behavioral responses in animal studies and improves memory issues in elderly patients. The purpose of this review is to analyze the molecular and genetics aspects of the EDR peptide effect on gene expression and synthesis of proteins involved in the pathogenesis of AD. The EDR peptide is assumed to enter cells and bind to histone proteins and/or ribonucleic acids. Thus, the EDR peptide can change the activity of the MAPK/ERK signaling pathway, the synthesis of proapoptotic proteins (caspase-3, p53), proteins of the antioxidant system (SOD2, GPX1), transcription factors PPARA, PPARG, serotonin, calmodulin. The abovementioned signaling pathway and proteins are the components of pathogenesis in AD. The EDR peptide can be AD.

## 1. Introduction

Understanding the etiology and progression of sporadic neurodegenerative diseases (NDDs) is an urgent problem of neuromedicine and neurobiology. Alzheimer’s disease (AD) is considered the most common NDD [1,2]. The disease is more frequent in older persons. The AD pathogenesis is associated with damage to hippocampus—a part of the limbic system, responsible for processing spatial information, forming emotions and consolidating memory. Diffuse amyloid plaques, surrounded by intracellular neurofibrillary tangles, formed by hyperphosphorylated τ-protein, discovered in animal models and post mortem brain studies, are the distinctive neuropathological features of AD [3,4]. Early manifestation of AD revealed dominant mutations in the β-amyloid precursor protein (APP) gene and presenilin 1 and 2 (PSEN1 and PSEN2) gene, which encode the γ-secretase components [4].

Currently, there are no known prevention methods for the progression of neurodegeneration in AD. In some cases, conservative treatment methods may slow down the development of its symptoms. Drugs used for the treatment of brain pathology, including those in elderly and senile people, belong to different pharmacological groups. Antioxidants, nitric oxide blockers, substances that suppress lipid peroxidation processes, etc., are among them. Peptide bioregulators appear to be a promising group of neuroprotectors due to their high physiological activity and absence of side effects [5,6,7].

Positively-charged short peptides rich in arginine were previously established to have neuroprotective properties [8]. In addition to excitotoxicity reduction, arginine-rich peptides also possess the ability to diminish mitochondrial dysfunction and inhibit the activation of extracellular matrix metalloproteinases in neuropathology, thus increasing the viability of the neurovascular unit in the brain in various pathological processes [9]. Changing the composition and sequence of amino acid residues in peptides rich in arginine will enable obtaining peptides with targeted neuroprotective action, potentially effective in AD and other NDDs [10].

**Figure 1:**
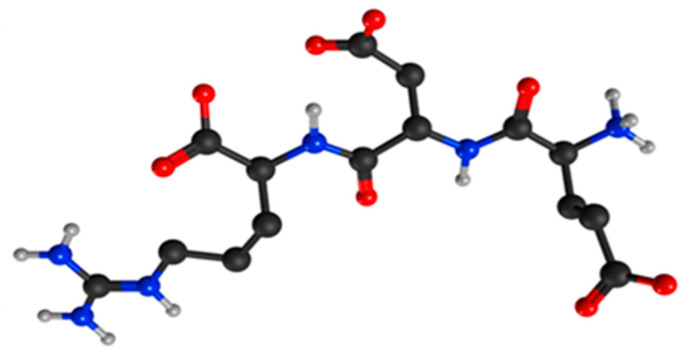
Spatial image of the EDR peptide structure. Oxygen atoms are marked in red, hydrogen atoms are marked in light gray, carbon atoms are marked in dark gray, and nitrogen atoms in blue.

EDR (Glu-Asp-Arg, Pinealon) (Figure 1), a tripeptide isolated from the polypeptide neuroprotective drug Cortexin, is one of the neuroprotective arginine-containing peptides. Oral administration of Pinealon revealed its effectiveness in correction of cerebral dysfunctions in older age groups. The EDR peptide contributed to neuronal apoptosis reduction, improvement of memory, attention and cognitive functions, acceleration of perceptual-motor responses, increase of mental performance, and decrease of the central nervous system (CNS) aging in the elderly [11,12,13].

Oral administration of Pinealon in addition to standard therapy in 72 patients with traumatic brain injury consequences and cerebrasthenia led to improved memory, reduced duration and intensity of headaches, emotional balance, and enhanced performance efficacy [11]. In patients with long-term consequences of traumatic brain injury, oral administration of the EDR peptide resulted in a decreased number of errors during the correction test. These patients manifested a significant increase in the α-index when determining the bioelectric activity of the brain. Thus, the EDR peptide stimulated neuroplasticity and integrative function of brain neurons after traumatic brain injuries [13].

The EDR peptide normalized the functional activity of the central nervous system in an experimental prenatal hyperhomocysteinemia model in rats. In cerebellar granule cell cultures, the EDR peptide increased the lag phase of MAP kinase activation and decreased the level of reactive oxygen species (ROS) [13,14,15].

Formation of synaptic contacts on the dendritic tree is an essential component of the neural network functioning. Disorders in the dendritic tree morphology, changes in the number and shape of spines are the signs of AD and other NDDs [16,17]. Mushroom-shaped spines form active synapses and are representative of a highly integrated neural network, which serves as the basis for learning and memory [18]. Restoration of the dendritic spine morphology was chosen as the criterion for evaluating the neuroprotective effect of the EDR peptide in the culture of hippocampal neurons in AD modeling in mice in vitro. The EDR peptide prevented the loss of neuronal mushroom-shaped spines in AD [19].

The purpose of this review is to analyze the molecular aspects of the neuroprotective activity of the EDR peptide in AD. To achieve this goal, the role of some molecules (MAPK/ERK, caspase-3, p53, SOD2, GPX1, PPARA, PPARG, serotonin, calmodulin) in the AD pathogenesis was analyzed. These data were compared with the regulation of gene expression and synthesis of the above molecules by the EDR peptide. Such an approach is required in order to identify the targets of the EDR peptide at early stages of AD.

## 2. EDR Peptide: Possible Molecular Aspects of the Regulation of Gene Expression and Protein Synthesis Involved in the Pathogenesis of Alzheimer’s Disease

### 2.1. MAPK/ERK Signaling Pathway: Role in the Pathogenesis of Alzheimer’s Disease, Regulation by the EDR Peptide

Mitogen-activated protein kinases (MAPKs) are evolutionarily conserved multifunctional signaling molecules, which play a key role in converting extracellular signals into intracellular responses. The most studied among them are four mammalian MAPK kinases: extracellular signal-regulated kinase 1 and 2 (ERK1/2), c-Jun N-terminal kinase (JNK), p38, and ERK5 [20].

The ERK1/2 cascade plays a central role in signal transmission from a wide variety of extracellular agents, which act through various receptors. In most cases, activation of these receptors is mediated by several mechanisms via Ras protein to cell membranes. The activated Ras protein recruits components of the MAPK cascade (Raf-1, B-Raf) to the plasma membrane and triggers their activation. Stimulation of ERK1/2 is followed by phosphorylation of substrates, responsible for proliferation and differentiation, morphology and plasticity of neurons, stress response control, and regulation of apoptosis [21]. Impairment of the signaling cascade regulation may result in NDDs, diabetes mellitus, and tumors [22].

The role of the MAPK pathway in the metabolic disorder of β-amyloid (Aβ), phosphorylation of the τ-protein, and regulation of inflammatory reactions in AD has been established [23,24]. When a sublethal concentration of the Aβ42 peptide is added to the culture of neurons, inhibition of the MAPK/ERK and PI3K/Akt pathways occurs, which leads to mitochondrial dysfunction, secretion of proinflammatory cytokines and cell death [25].

Neurofibrillary tangles are formed by hyperphosphorylation of τ-protein. It has been found that its phosphorylation is mediated by several kinases, including JNK, p38, and ERK5 [26].

Oxidative stress is a key risk factor for the AD development [27]. Under oxidative stress and AD development, free radicals activate the JNK and p38 signaling pathways [28]. Activated MAPK signaling pathways contribute to the pathogenesis of AD through various mechanisms, including induction of neuronal apoptosis [29,30], transcriptional and enzymatic activation of β- and γ-secretases [31], and phosphorylation and stabilization of the β-amyloid precursor (APP) [32,33].

Apoptosis signal-regulating kinase 1 (ASK1) is known to be a part of the MAPK family. It is activated in response to oxidative stress [34]. APP dimerization induces activation of the ASK1-MKK6-p38 signaling pathway, which leads to the phosphorylation of the τ-protein [35]. ASK1 forms a signaling complex with APP, MKK6, JNK1 and can induce apoptosis of neurons [36]. Aβ42 aggregates entail macrophage activation in brain tissues. Activated macrophages produce ROS and pro-inflammatory cytokines (TNF-α, IL-1β), which activate the MAPK signaling pathways. Under oxidative stress, activation of JNK and p38 occurs, which leads to stimulation of β-secretase gene expression. In this case, the ERK1/2 complex down-regulates the expression of β-secretase [37]. γ-secretase is activated by MEKK1, IFN-γ, IL-1β, TNF-α and blocked by a JNK inhibitor. The activation of γ-secretase through MEKK1 triggers a signaling pathway involving JNK kinase. TGF-β2 binds to APP and initiates APP-dependent apoptosis through JNK and caspase-3 activation [38].

Thus, MAPK signaling pathways can contribute to the pathogenesis of AD by regulating neuronal apoptosis, β- and γ-secretase activity, and phosphorylation of APP and τ-protein [39].

**Figure 2:**
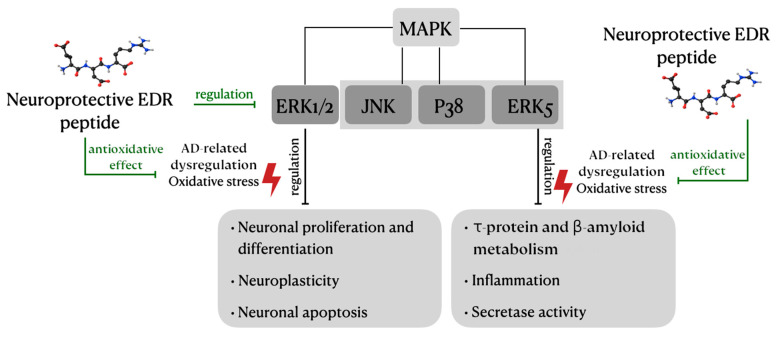
Possible pathway of the antioxidant and neuroprotective action of the EDR peptide in Alzheimer’s disease, associated with impaired MAPK signaling. The EDR peptide can exert neuroprotective and antioxidant effects in AD by regulating the expression of ERK1/2, which is evidenced by experimental data. Kinases JNK, p38, ERK5 are involved in the regulation of oxidative stress in neurons. Currently, the effect of the EDR peptide on these kinases has not been studied. However, it can be assumed that the antioxidant properties of the EDR peptide are associated with direct or indirect regulation of the functions of JNK, p38 and ERK5.

The EDR peptide decreased ROS synthesis caused by the receptor-dependent (ouabain, homocysteine) and non-receptor (hydrogen peroxide) activators of oxidative stress in granular cells of rat cerebellum. The ability of the tripeptide to reduce the production of ROS during an inflammatory reaction has been demonstrated in zymosan-activated neutrophil cultures [14]. ROS have been established to function as secondary messengers, triggering cascades of cellular signaling—the MAPK-ERK1/2 pathway, in particular [40,41]. The effect of the EDR peptide on the ERK1/2 level in neurons under the action of homocysteine was studied. In control neuronal cultures, addition of homocysteine led to ERK1/2 activation within 2.5 min, while in the presence of homocysteine and EDR tripeptide, an increase in the level of the ERK1/2 active forms occurred 20 min later. Thus, EDR has an inhibitory effect on ERK1/2 activation in rat cerebellar granule cells exposed to homocysteine. The neuroprotective effect of the EDR peptide is accompanied by a delayed ERK1/2 activation and a change in the onset of the cellular cycle phases. The limitation of ROS accumulation and cell death occurred at lower concentrations of the EDR peptide, while higher concentrations of the EDR peptide resulted in the modulation of the cellular cycle [14]. Thus, the EDR peptide is capable of exerting neuroprotective and antiapoptotic effects through the MAPK/ERK signaling pathway, thus preventing the AD development under oxidative stress conditions (Figure 2). ROS have been implicated in the activation of apoptotic processes also modulating others transcription factors, including phosphoinositide 3-kinase (PI3K)/Akt, nuclear factor (erythroid-derived 2)-like 2 (Nrf2), Kelch like-ECH-associated protein 1 (Keap1), and nuclear factor-κB (NF-κB). It is possible that the antiapoptotic effect of the EDR peptide is associated with the regulation of (PI3K)/Akt, Nrf2, Keap1, NF-κB; however, this hypothesis requires further investigation and experimental confirmation. The KE (Lys-Glu) dipeptide, which has antioxidant and antiapoptotic properties, was previously found to regulate the synthesis of NF-κB and p53 proteins in animal and human skin fibroblasts during their replicative senescence. Peptides KE, AED, KED, EDL, AEDG were also shown to reduce the expression of pro-apoptotic proteins caspase-3, p53 in various types of cells during replicative senescence [42,43,44,45,46]. It can be assumed that the regulation of apoptosis and antioxidant status of cells by di-, tri-, and tetrapeptides has both common features and differences depending on the type of cells and peptide structure. In this case, the neuroprotective properties of the EDR peptide, manifested in the regulation of apoptosis and synthesis of proteins of the antioxidant system, are one of the general patterns of peptide regulation.

### 2.2. Antioxidant System Proteins SOD2, GPX1: Role in the Pathogenesis of Alzheimer’s Disease, Regulation by the EDR Peptide

Brain neurons that actively consume oxygen have complex enzymatic and non-enzymatic defense mechanisms against the development of oxidative stress. Superoxide dismutase (SOD) and peroxiredoxins effectively compensate for oxidative changes in various subcellular compartments of neurons [47]. SOD is a key enzyme involved in superoxide radical detoxification. SOD2 is located in mitochondria and represents the only ROS-regulated isoform of this enzyme. The SOD2 concentration in the cerebral cortex of Tg2576 mice (AD model) increases with age [48]. It is possible that the increase in SOD2 expression in brain neurons during aging is a compensatory mechanism aimed at reducing the consequences of an increased oxidative stress, accompanying mitochondrial dysfunction. Mice lacking SOD2 synthesis develop neuropathology due to neuronal apoptosis resulting from mitochondrial oxidative stress [49]. Decreased SOD2 expression in the brain neurons of transgenic mice, carrying mutations in the amyloid precursor protein APP, is the cause of AD [50].

Modulation of SOD2 functions in the presence of amyloid plaques in transgenic mice with AD affects the endogenous oxidative stress level in mitochondria. Mice of a hybrid transgenic line with a deletion of one copy of the SOD2 gene and human APP/J20 genes manifested a faster and a more pronounced AD development [51]. A reduced synthesis of SOD2 leads to a decrease in the deposition of β-amyloid in the parenchyma and an increase in amyloidosis in the vasculature of the brain. Similar results were obtained in the AD model in Tg19959 mice, where the overexpression of mutant forms of β-amyloid in neurons was observed [52]. Another study evidenced the prevention of a long-term potentiation in cultured neurons by accumulation of amyloid peptide. This effect was reversed by the introduction of the MitoQ antioxidant and the SOD mimetic Euk-134 [53]. This is indicative of a synergistic effect of the mitochondrial oxidative stress and the progression of the amyloid plaque accumulation. It is possible that mitochondrial dysfunction and/or endogenous oxidative stress are the prerequisites for neuronal apoptosis in AD. Currently, no direct correlation between neuronal death and formation of amyloid plaques and neurofibrillary tangles has been found. However, a decrease in the bioenergetic capacity and mitochondrial metabolism of neurons may be a common precursor of these processes. This fact underlines the importance of minimizing oxidative damage in the brain to prevent neuronal apoptosis during AD development.

The role of glutathione peroxidase (GPx) in neuroprotection is also of great importance. GPx is a family of selenium-dependent enzymes, which catalyze the reduction of hydrogen peroxide, organic hydroperoxide and lipid peroxide by reducing glutathione, thus protecting the cells from oxidative damage. Cytosolic glutathione peroxidase (GPx1) is expressed in tissues with a high level of oxidative stress [54]. Patients with AD were found to have low erythrocyte GPx activity. It is possible that a decrease in the GPx function represents an integral disruption of the antioxidant system activity and can spread to brain neurons [55].

The EDR peptide has been discovered to reduce the level of hydroperoxides, exhibiting the ability to directly neutralize the primary products of lipid peroxidation. The latency period before the oxidation development extends in proportion to the increase in the EDR concentration. Cells isolated from the cerebellum of the EDR-treated rats were more resistant to oxidative stress [14]. Hypoxia was discovered to result in a three-fold increase in the initial ROS level. Against the background of a high ROS level, NMDA (N-methyl-D-aspartic acid) did not cause any additional increase in the amount of free radicals in the offspring of rats subjected to hypoxia [56]. A decreased amount of free radicals was evidenced in the neurons of the hypoxic rat offspring, treated with the EDR peptide. These data provide an indication of the EDR peptide ability to protect neuronal cells from the excitotoxic effect of NMDA. Thus, the action of the EDR peptide is mediated by an increase in the activity of neuronal antioxidant enzymes [57]. The effect of the EDR peptide on the activity of antioxidant enzymes in the brain of resistant and hypoxia-sensitive rats was studied. The SOD2 and GPx1 activity in the brain tissue of hypoxia-resistant rats was 2 times higher than in hypoxia-sensitive animals. Administration of the EDR peptide to hypoxia-sensitive animals led to an increase in the SOD2 and GPx1 activity in the brain to the level of hypoxia-resistant animals [57]. The results of the study indicate that in hypoxia-resistant rats, high activity of the antioxidant enzymes SOD2 and GPx1 provides protection against hypoxic effects and requires no additional stimulation with the EDR peptide. Thus, it can be assumed that the EDR peptide is capable of exerting a neuroprotective effect in AD by increasing the activity of the antioxidant enzymes SOD2 and GPx1 in brain neurons.

### 2.3. Caspase-3 and p53 Protein: Role in the Pathogenesis of Alzheimer’s Disease, Regulation by the EDR Peptide

The p53 transcription factor plays an important role in responding to DNA damage, genome integrity maintenance and suppression of tumor development [58]. Genes controlled by the p53 protein regulate a number of biological processes. Disruption of their expression and p53 activity leads to the development of NDDs, cancer, and metabolic syndrome [59]. The p53 protein, together with the p63 and p73 transcription factors, regulates the cell cycle, apoptosis, differentiation, and cell aging [60].

In sporadic and familial forms of AD, overexpression of the p53 protein was revealed in the cortex neurons of the frontal and temporal lobes, glial cells of the cortex and white matter of the brain [61]. Increased expression of the p53 protein was also found in the hippocampus of AD mice. In some cases, an increase in p53 expression correlated with the accumulation of Aβ42 peptide in brain neurons of animals and humans with AD [62].

Under normal conditions, the transcription factor p53 migrates to the internal mitochondrial matrix in response to DNA damage. The mitochondrial p53 protein forms an inhibitory complex with Bcl2 and Bcl-xL, which leads to the release of cytochrome C from mitochondria into cytosol and activation of caspases [63]. Translocation of p53 into mitochondria changes the mitochondrial membrane potential. Peptide Aβ42 enhances the expression of p53 in brain neurons in AD [64].

Low levels of basal oxidative and nitrosative stress in brain neurons correlated with low expression of the p53 gene in mice. This was accompanied by a decrease in the DNA damage, accumulation of lipid peroxidation products and carbonylated proteins, a weakening of protein nitrosylation, and an increase in the antioxidant enzymes activation. It is possible that pharmacological inhibition of the p53 prooxidant activity can inhibit neurodegeneration in AD [65]. Thus, the p53 protein deficiency reduces oxidative stress [66].

Proapoptotic caspases are assumed to contribute significantly to the progressive death of neurons in AD [67].

The activation of the main effector caspase-3 leads to the development of neurodegenerative processes, associated with chronic and acute disorders of cerebral circulation. The involvement of caspase-3 in the regulation of synaptic plasticity has been described [68]. Caspase-3 takes part in the APP processing into amyloidogenic fragments. In this regard, the accumulation of caspase-cleaved APP can be considered as an early stage of AD pathogenesis [69,70]. An increase in the active caspase-3 level in the axons of the brain hippocampal neurons in AD is localized at the formation sites of neurofibrillary tangles and plaques. Caspase-3 is activated in synapses in response to neuronal apoptosis [71].

In addition to the classical role of caspase-3 in the activation of neuronal apoptosis, this enzyme has been revealed to participate in the regulation of synaptic plasticity and metabolism of the τ-protein in AD [72,73]. By cleaving serine-threonine kinase Akt, Caspase-3 activates the GSK3β kinase pathway, which regulates the τ-protein phosphorylation. Pharmacological blockade of caspase-3 activation in the central nervous system can prevent the phosphorylation of the τ-protein. Drugs aimed at inhibiting caspase-3-dependent Akt cleavage may be promising for the prevention of τ-protein metabolism disorders in AD.

The effect of the EDR peptide on the caspase-3 synthesis in brain structures and the correlation of this process with the ability to learn in rats of different ages in an experimental model of acute hypoxic hypoxia were studied. The EDR peptide reduced the expression of caspase-3 in the brain and improved the learning indices in the Morris maze in young and old animals [74]. In another study, the administration of the EDR peptide led to the activation of the caspase-3 protease in the cerebral cortex and brain stem structures of old rats while maintaining the level of expression of active caspase-3 in the brain at the control level [74].

The intensity of caspase-dependent apoptosis of neurons is known to decrease in age-associated cerebral ischemia. At the same time, the pro-inflammatory status of the brain structures increases [75]. Old rats with acute hypoxic hypoxia manifested an increase in the activity of caspase-3 in the brain neurons. The introduction of the EDR peptide prevented the activation of caspase-3 in neurons [12]. The activity of caspase-3 in the brain decreases with ageing, while an increase in its activity may occur against the background of neurogenesis activation [76]. Thus, the EDR peptide may have a neuroprotective effect in AD by regulating proapoptotic factors: caspase-3 and p53 protein.

### 2.4. Transcription Factors PPARA, PPARG: Role in the Pathogenesis of Alzheimer’s Disease, Regulation by the EDR Peptide

Inflammation plays an important role in the pathogenesis of AD [77]. The Aβ42 peptide-stimulated expression of pro-inflammatory genes in the myeloid clone cells is antagonized by the action of nuclear ligand-activated hormone receptors family—peroxisome proliferation-activating receptors (PPARs). PPAR-α agonists were found to inhibit the Aβ42 peptide-stimulated expression of cytokine genes (TNF-α, IL-6, TNF-α) by blood monocytes and macrophages. The PPAR-α agonist (WY14643) inhibits macrophage differentiation and COX-2 gene expression. Thus, PPAR-α inhibits monocyte-mediated inflammatory responses [78,79,80].

Microglia or monocytes interaction with β-amyloid fibrils in AD activates a signaling cascade involving tyrosine kinase, which leads to the stimulation of gene expression of pro-inflammatory cytokines [81]. At the same time, the expression of the PPARα gene in the brain neurons in AD decreases [82].

The presence of extracellular amyloid plaques containing Aβ42 peptides, which occur as a result of amyloidogenic proteolytic processing of APP by β- and γ-secretases, is one of the pathogenetic signs of AD. APP can also be cleaved in a non-amyloidogenic way aided by α-secretase, preventing the formation of Aβ42 peptides. Therefore, mutations in the prodomain of disintegrin α-secretase and metalloproteinase-10 (*ADAM10*) are associated with an enhancement in the Aβ42 synthesis and an increased probability of AD development. PPAR-α activation was found to induce proteolysis of APP by α-secretase in hippocampal neurons by inducing *ADAM10* gene transcription [83]. It has been shown that PPAR-α can modulate APP processing and Aβ42 peptide formation. 5xFAD mice with a *PPARα* gene knockout, revealed a decreased life span in the AD model, which correlated with a high concentration of Aβ42 peptide in the hippocampus. It was found that increased expression of the Aβ42 peptide is associated with a shorter lifespan in AD patients [84]. Thus, the PPAR-α protein can contribute to the reduction of the inflammatory response severity and the accumulation of the synaptotoxic peptide Aβ42 in AD.

PPARG is the target of several pharmacological agents, regulating metabolism, immune response and neuroplasticity. At an early stage of AD, activation of PPARG expression can inhibit the disease progression [85].

Epidemiological studies have shown that the use of non-steroidal anti-inflammatory drugs (NSAIDs) can reduce the risk of AD. This effect is due to the ability of NSAIDs to activate PPARG and suppress inflammatory responses in the brain of AD patients [86]. It has been established that the PPARG gene plays an important role in modulating the formation of β-amyloid during inflammation. This suggests that the protective mechanism of NSAIDs in AD may include PPARG activation and a decrease in the transcription of the gene for the enzyme that cleaves the amyloid precursor protein (BACE1) [87]. Thus, the PPARG gene is a potential target for AD pharmacotherapy.

The EDR peptide was established to increase the expression of the PPARA, PPARG genes in humans under stress conditions, induced by increased physical activity. The EDR peptide contributed to the normalization of the number of spines of neuron dendrites obtained from 5xFAD mice with AD and the PPAR-α gene knocked out [19,84]. 

**Figure 3:**
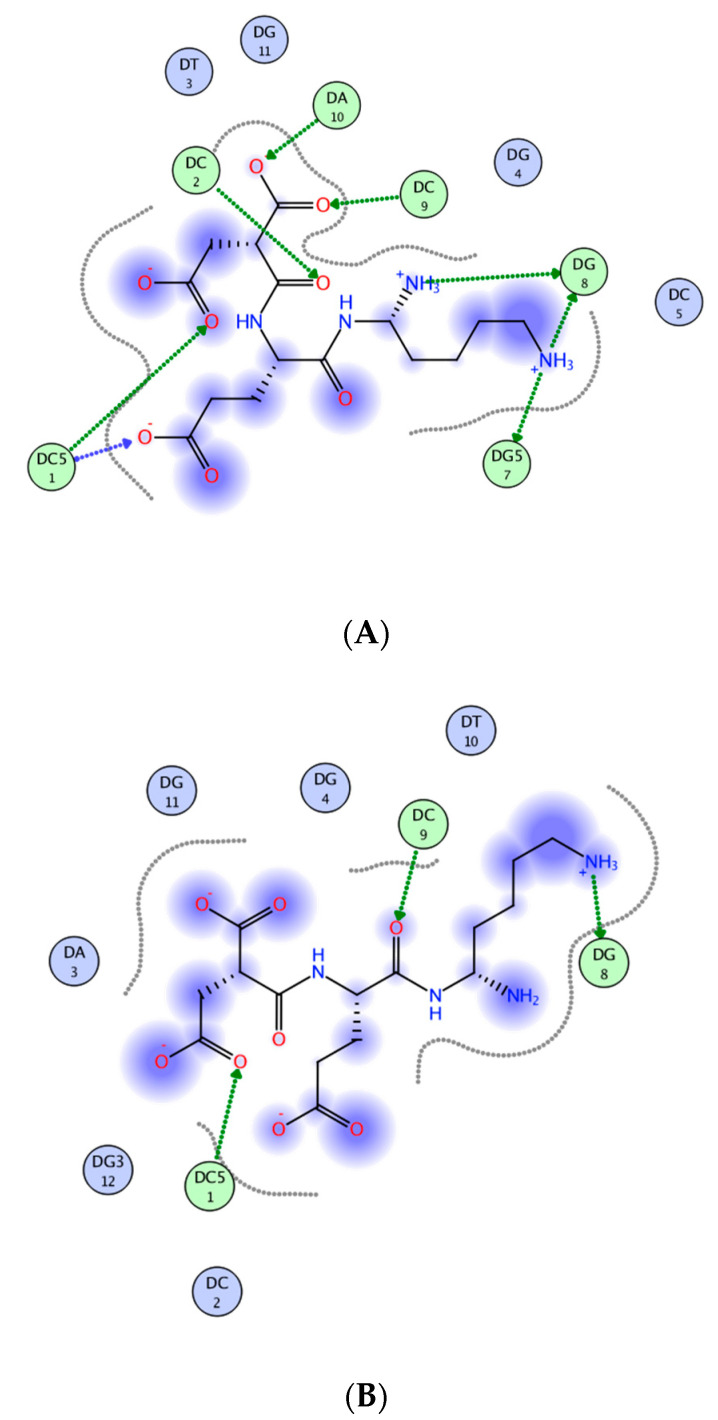
Diagram of contacts between the EDR peptide and nucleotides: (**A**)—d(CCTGCC)_2_ sequences, (**B**)—d(CCAGC)_2_ sequences. The green arrow indicates the direction of proton transfer by the atom/to the atom of the peptide side chain. The blue arrow indicates the direction of proton transfer by the atom/to the atom of the peptide main chain. The dotted line indicates the ligand-solvent contact area (modified according to [88]).

Analysis of the promoter regions of the PPARA and PPARG genes indicates the presence of CCTGCC, CCAGCC binding sites for the EDR peptide [88]. Three possible binding sites for the EDR peptide and 5 sites for the PPARG gene were found in the promoter of the PPARA gene (Table 1, Figure 3). In addition, the EDR peptide is assumed to interact with CG sites in the promoters of various genes [89].

It is possible that the neuroprotective effect of the EDR peptide is due, among other factors, to the regulation of the expression of the PPARA and PPARG genes.

## 3. Serotonin: Physiological Role in the Pathogenesis of Alzheimer’s Disease, Regulation by the EDR Peptide

Serotonin (5-hydroxytryptamine) is one of the main neurotransmitters that regulate brain function. Tryptophan hydroxylase (TPH) catalyzes the rate-limiting step of serotonin biosynthesis from 5-hydroxy-l-tryptophan (5-HTP) [90,91]. The extract of the *Griffonia simplicifolia Baill* has been found to contain large amounts of 5-HTP. This, according to the authors of this study, makes it possible to recommend the specified plant extract for the complex treatment of neurodegenerative diseases associated with impaired serotonin synthesis [90]. In addition, there is evidence that tryptophan metabolites may have neuroprotective effects in AD. Tryptophan metabolites 5-hydroxyindole-acetic acid and kynurenic acid modulate the activity of neuronal matrix metalloproteinase (neprilysin). Neprilysin activation promotes biodegradation of toxic Aβ42 peptide and reduces the severity of AD manifestations [92].

Serotonin is used to synthesize N-acetylserotonin (NAS), a melatonin precursor, which produces a geroprotective effect on brain cells [93,94]. In AD, a decrease in the concentration of serotonin in the anterior cortex, hippocampus, amygdala, and striatum occurs. Impaired serotonergic regulation may lead to a deterioration in cognitive function in AD [94,95].

Serotonin regulates neuronal axonal growth, synaptogenesis, and dendritic spine formation. These processes ensure the formation of behavioral reactions, regulation of mood and body temperature, appetite, sleep, and cognitive functions [96]. Dysfunction of serotonergic signaling leads to amyloidogenesis, hyperphosphorylation of the τ-protein, and the formation of neurofibrillary tangles characteristic of AD [97,98,99]. The accumulation of TPH and its oxidation products, resulting from a decrease in the transportation of TPH to axon terminals, may contribute to the degeneration of serotonergic neurons in AD [95]. The use of selective serotonin reuptake inhibitors in AD mice decreases the synthesis of Aβ42 peptide and the formation of senile plaques [100].

It was found that EDR peptide increased serotonin synthesis in the neuronal cultures of cerebral cortex in rats [88]. One possible binding site for the EDR peptide was discovered in the TPH1 gene promoter regions [88] (Table 1, Figure 3). There is evidence that the use of a plant extract containing TPH and some metabolites of tryptophan, required for the serotonin synthesis, have a neuroprotective effect [90,92]. Based on these data, it can be assumed that the EDR peptide, which activates the serotonin synthesis in neurons, affects one (associated with THP) or several stages of serotonin synthesis from tryptophan, which may explain its neuroprotective effect in case of AD. The determination of the targets of action of the EDR peptide in the biochemical cascade of serotonin synthesis is an important aspect of further study on the mechanism of action of this peptide.

## 4. Discussion

Ultrashort peptides (2–4 amino acid residues) are signaling molecules involved in the homeostasis regulation at various organismal levels. Long-term studies have revealed the selective nature of short peptides’ activity (tissue- and gene-specific) [101]. To explain the target mechanism of peptide regulation of gene expression and protein synthesis, models of the interaction of short peptides with DNA and histone proteins have been proposed [102,103]. These models provide means for the explanation of the high biological activity of ultrashort peptides. A model for the pathological processes development, according to which the disturbances in peptidergic regulation play a key role in the development of the said processes, has been proposed. Correction of such disturbances by the administration of short peptides may result in the pathological process’ regression and normalization of the body functions. High biological activity and tissue specificity, as well as the absence of species-specificity and immunogenicity, are the advantages of ultrashort peptides [104,105].

It was established that the EDR peptide possessed neuroprotective properties when administered orally in patients with traumatic brain injury and cerebrosthenia, as well as in experimental neuropathology induced by hypoxia and oxidative stress in AD and Huntington’s disease models in vitro. Presumably, the molecular mechanism of the EDR peptide biological activity, like a number of other short peptides, is associated with its ability to penetrate into the cytoplasm and the cell nucleus [106] and regulate gene expression and synthesis of the corresponding proteins [88,103,104].

In vitro studies have shown that the EDR peptide interacts with DNA, exerting a destabilizing effect on the secondary structure of a macromolecule and a compacting effect on the volume of its molecular coil [106]. Molecular modeling suggested two possible binding sites for the EDR peptide: d(CCTGCC)_2_ and d(CCAGC)_2_ (Figure 3). Binding sites for the EDR peptide were found in the promoter regions of genes, encoding proteins which regulate the functional and antioxidant neuronal activity (PPARA, PPARG, SOD2, GPX1, TPH1) [88] (Table 1). The EDR peptide was established to reduce the severity of neuronal apoptosis, determined by the caspase-3 and protein p53 synthesis, and possess antioxidant properties.

## 5. Conclusions

**Figure 4:**
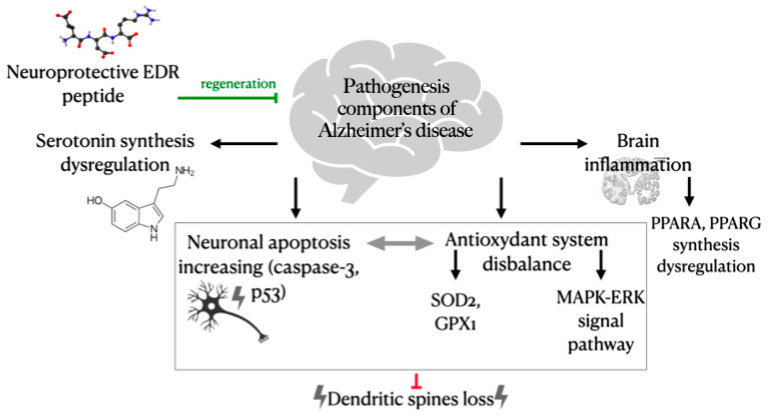
Possible mechanism of the EDR peptide neuroprotective effect on the main components of pathogenesis in Alzheimer’s disease.

Analysis of the literature data and the authors’ research results has allowed establishing the pathogenetic components of Alzheimer’s disease, which may be influenced by the neuroprotective peptide EDR (Figure 4).

In AD, the following disturbances are observed: impaired serotonin synthesis, imbalance of the antioxidant system, which involves the signaling pathway MAPK-ERK, SOD-2, GPX1, activation of neuronal apoptosis through caspase-3 and p53 protein (possibly due to oxidative stress), development of an inflammatory reaction in the brain, characterized by the dysfunction of PPARA and PPARG transcription factors. This results in the loss of dendritic spines of neurons in the brain, which is one of the main morphological signs of AD and the cause of cognitive impairment. The EDR peptide produces a protective effect on all the listed components of the AD pathogenesis and prevents the dendritic spines loss in hippocampal neurons. Thus, the EDR peptide is a promising neuroprotective agent, potentially effective in the early stages of AD.

## Figures and Tables

**Table 1:** Possible EDR peptide binding sites in promoters of genes, encoding neuroprotective proteins.

Gene	Gene Regulatory Site, Range -499 to 100 bp (cDNA 5′→3′)	Gen Bank NO
PPARA *Homo sapiens*	ACCGGCTCATCGCACAGAGTAGCAGAGCCGGGCTCATCGAGGAGGCAGGAGGGGCTCGCCAGCGTGGCACGGGCGCCCGGCGGGAACCTCCACCCGCCCCGCGGCCGCGCGTCCCCGCCTCGAATTCAGCCCCGCCCCGGTGCGCCGGGCTGGAGGGGCGCTGACGCTCAGCGGTGTCCCATCGGTGACCTTGGACGGTCCCTCCACCTCTCCGGCCTCAGTTTCCCTTGGCTGCAGCGGCCGCGGGGCGCTAGGTGGGAGCCGCTGAGCGCTCCCGGGGCCCCGCCCACCGCGAGCAGCCAATCGGGCGCCGCCCTCCGGGGGGTGTGTCCCGGGGCCGAGGCCCGGGGCCCGGAGGGCGCGCGGGGCGGGCGGGGCTTCCGGGTCGGGCCTCGGGACACTGGCTCGCGCGGACCGGGGCAGGGGGCGGGCCGAGGGGCGGTGCGTGTCGCGGGGGCGCGGCTGGCACGGACGCGCGGAGGCGGCGCCGGGCATGGGCCGTGGACGCGGCGGCCCCGCGGCGGGGGCAGCGGGCGGCGGGGGCGGAGGCGGCCGCTAGCGCCCTGCCCGGCGCCGCCTCCTTCGGCGTTCGCCCCACGG	NM_005036.4
PPARG *Homo sapiens*	ACCAAGGGACCCGAAATATGCTTTAATTAAATTTTCTTTTAAAATGTCACTGGAAAGAACATCTTGGGAAGACGGCCTGGCCGATCGCCGTGTGAAGGGCAAGCCACTCTGGCCGAGAGGGAGCCCCACACCTCGGTCTCCCCAGACCGGCCCTGGCCGGGGGCATCCCCCTAAACTTCGGATCCCTCCTCGGAAATGGGACCCTCTCTGGGCCGCCTCCCAGCGGTGGTGGCGAGGAGCAAACGACACCAGGTAGCCTGCCGCGGGGCAGAGAGTGGACGCGGGAAAGCCGGTGGCTCCCGCCGTGGGCCCTACTGTGCGCGGGCGGCGGCCGAGCCCGGGCCGCTCCCTCCCAGTCGCGCGCCGCCGCCCCCGCCCCCGCCCCCGCCCCCGCCCCCACCCCCACCCCCACCCCCACCCCCAGCCGGCGCCCGCGCCCGCCCCCGCGCCGGGCCCGGCTCGGCCCGACCCGGCTCCGCCGCGGGCAGGCGGGGCCCAGCGCACTCGGAGCCCGAGCCCGAGCCGCAGCCGCCGCCTGGGGCGCTTGGGTCGGCCTCGAGGACACCGGAGAGGGGCGCCACGCCGCCGTGGCCGCAGGTC	NM_138712.3
TPH1 *Rattus norvegicus*	GCTTCTCCTATAAGAGGCGGCAGCTCCCGTCCGCAGGTGACCCTCTGAACTCCAGTGGCTTTGAGGTCCTCTTTCCAGTGCCGGATCCTGCCCACTGGGTCATCTTCATTCAGATTCACCATGATTGAAGACAACAAGGAGAACAAAGACCATTCCTCAGAAAGGGGGAGAGTGACTCTCATCTTTTCCTTGAAGAATGAAGTTGGAGGACTCATAAAAG	X53501.1
GPX1 *Homo sapiens*	GACTCTGCCCGGTTAGAAAACCCGCACGAGGGCGGTGCCGCTTTGGAGACAGGGAGGAGGGAGACCGGAAGCCTAGATCCCTCTGGCTGTCCCCTGCACTGCCGGTAACATGGCACAGGAGAGGAGGGCTGTTTGTGCACGGGCAGCTCCTGCAGCTGCTGCCGTCGCCCACCAGCCTCCTATGCCAAACCCCACATCCTAACTCAGGAACCTCTGAGAAAAAACGGAGCCCTCGAGGGCCCCAGCCCTTGGAAGGGTAACCTGGACCGCTGCCGCCTGGTTGCCTGGGCCAGACCAGACATGCCTGCTGCTCCTTCCGGCTTAGGAGGAGCACGCGTCCCGCTCGGGCGCACTCTCCAGCCTTTTCCTGGCTGAGGAGGGGCCGAGCCCTCCGGGTAGGGCGGGGGCCGGATGAGGCGGGACCCTCAGGCCCGGAAAACTGCCTGTGCCACGTGACCCGCCGCCGGCCAGTTAAAAGGAGGCGCCTGCTGGCCTCCCCTTACAGTGCTTGTTCGGGGCGCTCCGCTGGCTTCTTGGACAATTGCGCCATGTGTGCTGCTCGGCTAGCGGCGGCGGCGGCGGCGGCCCAGTCGGTGTATG	NM_000581

The possible binding sites for the EDR peptide are highlighted in bold.

## Data Availability

Not applicable.
